# Pulsed Thermography Inspection of Composite Anticorrosive Coatings: Defect Detection and Analysis of Their Thermal Behavior through Computational Simulation

**DOI:** 10.3390/ma13214812

**Published:** 2020-10-28

**Authors:** Marcella Grosso, Isabel C. P. Margarit-Mattos, Gabriela R. Pereira

**Affiliations:** 1 Laboratory of Non Destructive Testing, Corrosion and Welding (LNDC), Department of Metallurgical and Materials Engineering, Federal University of Rio de Janeiro, Rio de Janeiro 21941-594, Brazil; margarit@metalmat.ufrj.br (I.C.P.M.-M.); gpereira@metalmat.ufrj.br (G.R.P.); 2 Department of Metallurgical and Materials Engineering, POLI and COPPE/UFRJ, Federal University of Rio de Janeiro, P.O, Rio de Janeiro CEP 21941-972, Brazil

**Keywords:** thermography, coating, computational simulation

## Abstract

The use of anticorrosive coatings has been a powerful method to be applied on the surface of metallic materials to mitigate the corrosive process. In this study, the focus is composite coatings that are commonly used on the internal surface of storage tanks in petrochemical industries. The development of non-destructive methods for inspection of faults in this field is desired due to unhealthy access and mainly because undercoating corrosion is difficult to detect by visual inspection. Pulsed thermography (PT) was employed to detect undercoating corrosion and adhesion loss of anticorrosive composite coatings defects. Additionally, a computational simulation model was developed to complement the PT tests. According to the experimental results, PT was able to detect all types of defects evaluated. The results obtained by computational simulation were compared with experimental ones. Good correlation (similarity) was verified, regarding both the defect detection and thermal behavior, validating the developed model. Additionally, by reconstructing the thermal behavior according to the defect parameters evaluated in the study, it was estimated the limit of the remaining thickness of the defect for which it would be possible to obtain its detection using the pulsed modality.

## 1. Introduction

Over decades, the use of protective coatings, both organic and inorganic, has been one of the more versatile techniques for corrosion control. Although the development of formula and new materials have given significant improvement in the coatings efficiency, during application or along service life, failures may develop and must be detected for maintenance [1,2]. Specially, for organic coatings, the degradation can be classified into two types of defect: cosmetic and that lead to corrosion [1]. One important defect is adhesion loss that may arise in consequence of problems in surface treatment, or during permeation of the coating by the corrosive media. Localized undercoating corrosion is an important failure to be detected as early as possible. However, in the early stages, failures cannot be detected by visual inspection. In this context, the nondestructive inspection becomes of great importance to be performed either after application of the coating, in order to ensure the application efficiency, as also periodically to monitor integrity during coatings service life [3].

Among the non-destructive techniques, thermography has gained great importance in different industry sectors, because it allows the inspection of large areas in a short time and it is also able to inspect various types of materials, such as: metals, composites and polymers. In addition to these advantages, the technique does not need physical contact with the material surface being inspected and is capable of detecting both surface and subsurface defects [4].

Thermography aims to analyze the thermal distribution on the material surface by an infrared camera. Based on the thermal excitation mode, the technique can be classified into: passive and active thermography. In active thermography, an external excitation source is required to induce a temperature difference (thermal contrast) between the defective region and the non-defective region. The type of thermal excitation that will be used determines the modalities of active thermography: for optical excitation, pulsed, pulsed phase and lockin; for electromagnetic excitation, pulsed eddy current thermography and for mechanical excitation, vibrothermography [4,5,6,7,8,9]. Different from other modalities, in pulsed thermography (PT) thermal excitation is used for a short time and its main interest is the monitoring of the thermal evolution on the material surface after heating. The result of this modality is presented in the form of a sequence of thermograms and areas of different thermal behavior as compared to the neighborhood can be correlated to the presence of defects [4,7,10,11,12,13].

Many studies have been recently developed to evaluate the potential application of thermography for non-destructive analysis of various materials, such as composites [10,11,12,14,15,16,17,18]. Specially for composite coatings, the literature is mainly dedicated to the use of thermography to estimate the coating thickness [19,20] and also for inspection of CUI (corrosion under insulation) for thermal barrier coatings (TBC). Analyzing the capacity of the technique to detect coatings disbondment, in the study developed by Ptaszek et al. [21], the response of three different forms of artificial disbondments was analyzed by PT. Zhao et al. [22] in their study showed that it was possible to correlate temperature distribution with thickness and also with microstructure change in TBC coating. The pulsed thermography was evaluated by Tang et al. [23] to analyze the influence of experimental parameters on the contrast obtained for TBC uneven thickness detection both experimentally and theoretically (through FEM simulation). Another study using the technique to estimate TBC thickness was performed by Bu et al. [24] and, unlike the others, he used the simulated annealing (SA) algorithm, based on the heat conduction inverse problem. The results obtained by pulsed thermography were compared to the ones obtained by eddy current and the relative error was less than 10%.

In this paper, pulsed thermography was employed to detect undercoating corrosion and adhesion loss of commercial anticorrosive coatings. It was also evaluated the influence of geometry, depth and type of filling of defects with iron oxide in their thermal behavior. Additionally, a computational simulation model was developed to reproduce the thermal behavior of the defects, allowing the analysis of the influence of the parameters related to the defects in the detection presented by the technique.

## 2. Experimental Study

### 2.1. Materials

Specimens with controlled defects were prepared representing failures of localized undercoating corrosion and adhesion loss. The defects had known dimensions, geometry and location. For this study, three samples were evaluated, called S1, S2 and S3. Each sample consisted of a carbon steel substrate (150 mm × 100 mm × 4.7 mm). Before applying the coatings, holes were machined with different areas in the substrate. In sample S1, three FBH (flat bottom holes) were inserted and in sample S2, three conical holes. In sample S3, two notches were inserted and a defect of adhesion was artificially made. The features and dimensions of the failures are in Table 1. In S1 and S2 samples all defects were filled with Fe_3_O_4_ to act artificially as an undercoating solid corrosion product.

**Figure 1:**
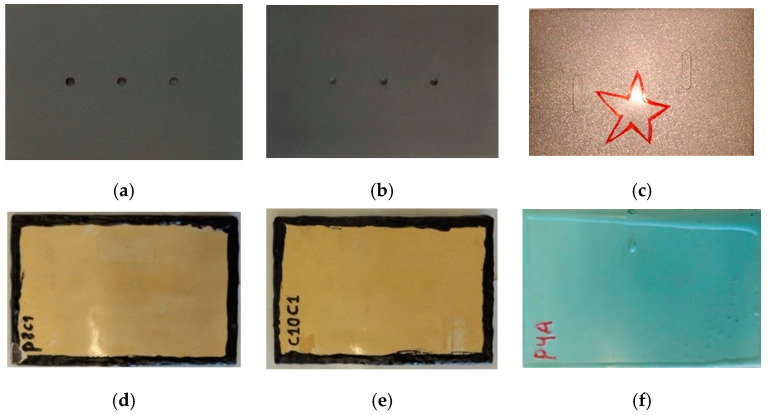
Visual aspect of the defects before the coating application for sample: (**a**) S1, (**b**) S2 and (**c**) S3 and visual aspect after the coating application of the samples (**d**) S1, (**e**) S2 and (**f**) S3.

After the insertion of defects, a thin layer of the coating, in liquid form, was applied, avoiding the area of the holes; and with this coating layer still wet, a free film of the same coating, already cured, was put onto the entire surface of the sample. In this process, the uncured coating layer acted as an adhesive for the film adhesion. Two commercial coatings were used on the samples: epoxy coating with glass flakes (samples S1 and S2) and epoxy with ceramic flakes (sample S3) (Table 2). Figure 1 shows the visual aspect of the samples before and after application of the coatings. According to these images, the presence of defects inserted at the metal surface was not observable by visual inspection.

### 2.2. Pulsed Thermography (PT) Experimental Tests

These results are presented in a sequence of thermograms in which the defective regions exhibited different temperatures in relation to non-defective regions of the sample.

**Figure 2:**
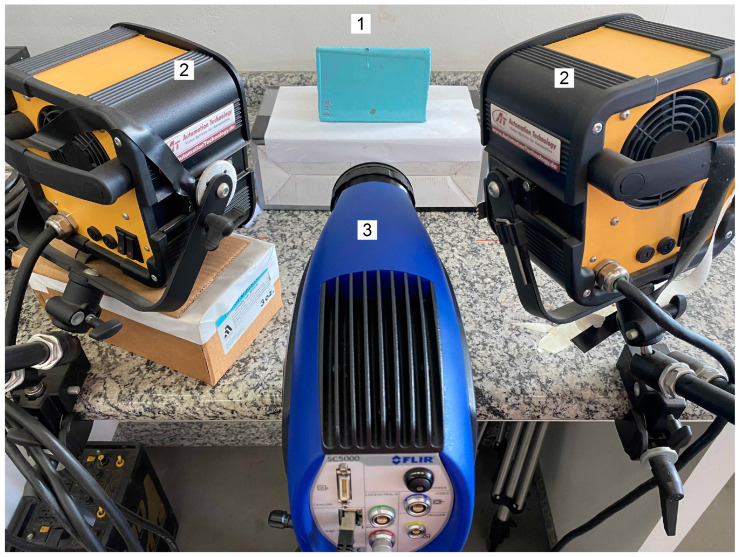
Photography illustrating the positioning of the equipment during the performance of the experimental tests: 1—sample, 2—halogen lamps and 3—infrared camera.

To carry out the experimental tests to inspect the specimens using PT, an infrared camera, which had a InSb detector, a thermal sensitivity of less than 30 mK and a resolution of 640 × 512, was used. Two halogens lamps of 5 kW controlled by an electronic module were used to generate a 10 s thermal excitation. The inspections were done on the reflection mode, as it is shown in Figure 2.

### 2.3. Data Processing and Image Post-Processing

The results of the inspections using PT are presented as sequences of thermograms. By analyzing this sequence, besides being possible to visually detect regions of different temperatures in the specimen, it is also possible to measure the thermal contrast between these regions. This parameter is extremely useful in extracting the defect information for quantitative analysis. For this study, the absolute thermal contrast, C^a^(t), was evaluated and corresponded to increased or excess temperature with respect to a reference region at a given time t. It can be calculated as follows (Equation (1)):

(1) $$C^{a}\;\left(t\right)=T_{\mathrm{def}}\;\left(t\right)-T_{\mathrm{ref}}\left(t\right)$$

where C^a^ is the absolute thermal contrast (°C), T_def_ is the temperature in the defective area and T_ref_ is the temperature in the reference region, without defect.

After calculating this parameter, the thermogram that represents the highest thermal contrast in the defect region is determined and thus, becomes representative for image post-processing.

## 3. Computational Simulation Model

The computational simulation model was developed using the finite element method in order to reproduce and calculate virtually all physical equations that describe the phenomena present during the PT experimental tests. The creation of this model becomes of great importance as it can be a useful tool for defining the defect detection limits of the technique in these materials, thus reducing costs related to the manufacture of samples and the use of inadequate equipment. Beyond the detection limit, the creation of this model may also help in determining the best parameters that will be chosen in the inspection.

This model was created and developed in Comsol Multiphysics, version 5.1 (Burlington, MA, USA). Although this software has several physical interfaces, there is no ready-made model capable of reproducing the pulsed thermography, i.e., the user becomes responsible for selecting the phenomena and adjusting the conditions of the model for each application.

**Figure 3:**
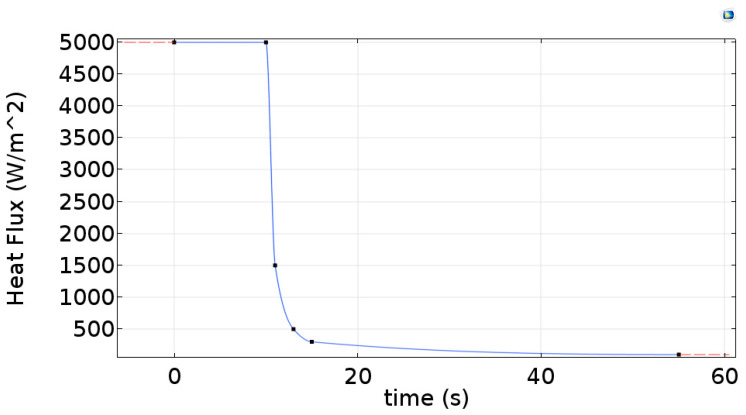
Heat flux incident function on the sample surface over time obtained.

The model was created through the heat transfer module in solids and the corresponding physical equations were inserted:

(2) $${\rho C}_{p}\frac{\partial T}{\partial t}=\;\nabla \times \left(k\nabla T\right)+\;Q$$

(3) $$-n\times \left(-k\nabla T\right)=\;\varepsilon \sigma \left(T_{\mathrm{amb}}^{4}-{\;T}^{4}\right)$$

(4) $$-n\times \left(-k\nabla T\right)=h\times \left(T_{\mathrm{amb}}-\;T\right)$$

where ρ is the density of material, $C_{p}$ the thermal capacity, k the thermal conductivity, T the temperature, Q the energy deposited on the surface of the sample, t the time elapsed during the test, ε the thermal emissivity of the material, T_amb_ the ambient temperature, σ is the Stefan–Boltzmann constant (5.7 × 10^−8^ W m^−2^ K^−4^) and h is the convective heat transfer coefficient in the air [25].

(5) $$-n\times \left(-k\nabla T\right)={\;q}_{0}$$

where $q_{0}$ is the heat flux incident on the sample surface due to the heating time. To obtain the function corresponding to this heat flow inserted in the sample surface that is not trivial, therefore, in this study, several simulations were carried out to analyze parameters that influence this function. Analyzing the results considering the heat flux and the heating time, it was decided to adopt a piecewise cubic interpolation, as shown in Figure 3. The main reason for this choice was based on the great similarity obtained in the cooling behavior of the lamps after the end of the heating period (from the time of 10 s on the horizontal axis of Figure 3).

During the 10 s heating time of the sample, the heat flow was 5000 W/m^2^ and after, the flux remains constant to 20 W/m^2^ up to the end of the inspection time.

**Figure 4:**
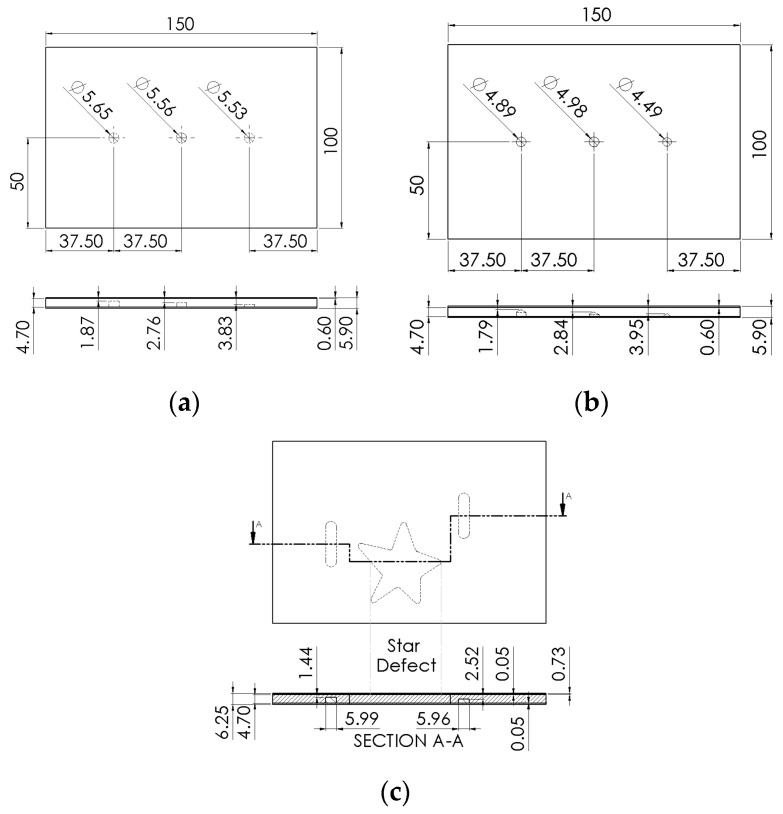
Virtual solid of the samples evaluated in this study, with the same geometric parameters of the samples used in the experimental tests: (**a**) sample S1, (**b**) sample S2 and (**c**) sample S3.

The virtual solid of each sample was constructed according to the geometry (substrate dimensions, coating and defects) of the actual samples. Figure 4 shows virtual solids of the samples evaluated in this study with the defects inserted.

The next step in the construction of the model was the choice of materials, including their main physical properties, which were used in each component of the samples to calculate the physical equations. Although each sample had a specific characteristic related to the type of defect and geometry, in all three samples were defined basically three components in their structure: substrate, coating and defects. The materials and their physical properties were obtained from the materials library of the software, as shown in Table 3 and also according to the literature.

For all simulations done, tetrahedral elements were used for the mesh construction and its refinement parameters were chosen based on both the processing time of the simulation and also on the resolution of the data in the areas of interest.

## 4. Results and Discussion

### 4.1. Pulsed Thermography (PT) Experimental Tests

Although PT results are presented in a form of thermogram sequence for each sample, for performing image post-processing analysis, only the highest thermal contrast thermogram of the sequence was chosen.

**Figure 5:**
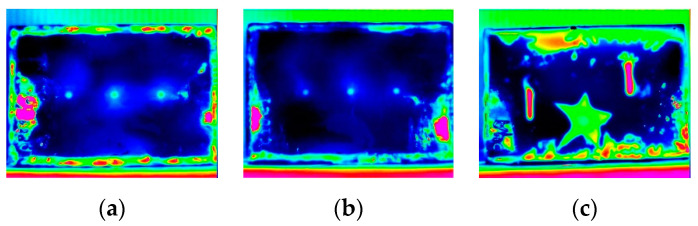
The highest thermal contrast thermogram obtained for: (**a**) S1, (**b**) S2 and (**c**) S3 samples.

For each test, the camera was configured for image acquisition at a 50 Hz frame rate and for 60 s monitoring. Thus, for each test, a sequence of approximately 3000 thermograms was obtained. Figure 5 shows the highest thermal contrast thermogram obtained for each sequence.

According to the results presented in Figure 5, all defects inserted in the samples were detected by PT: localized corrosion in samples S1 and S2, and the notches and adhesion failure with a star shape of 50 µm thickness between the substrate and coating in sample S3. Initially, we chose to evaluate two different types of coating in similar PT experiments to investigate if the chemical composition of the coatings would influence in the thermal response (behavior) of the samples. According to the results obtained (both regarding the thermograms in the images and the graph of the thermal evolution of the samples), the thermal behavior presented was very similar, which does not demonstrate an influence of the difference in chemical composition between the coatings. Comparing the thermograms obtained for S1 and S2, it was observed no significant change between the thermal contrast presented by the flat bottom holes (S1) and the conical bottom holes (S2). Another important aspect regarding samples S1 and S2 is that the Fe_3_O_4_ filling the holes did not influence their detection. This result is very interesting, since in the literature, defects are simulated with the presence of air and in the case of corrosion defects, the presence of iron oxide is mandatory and there are only few studies evaluating its influence in the detection of defects by different techniques. For sample S3, although both types of defects were detected, it can be observed that there is difference in relation to thermal contrast obtained for the thickness loss due to notches with red color and the star adhesion failure with green color. It seems that the thermal contrast can be considered an important parameter to help in the definition of the defect type.

### 4.2. Computational Simulation Results

**Figure 6:**
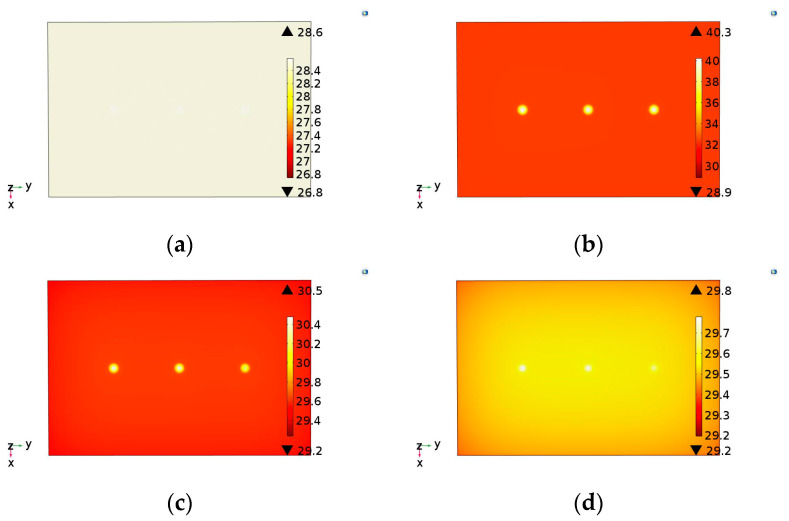
Images illustrating the temperature distribution on sample S1 obtained by the simulation model developed: (**a**) 0 s (at the moment that the lamps are turned on), (**b**) 10 s, (**c**) 20 s and (**d**) 30 s.

**Figure 7:**
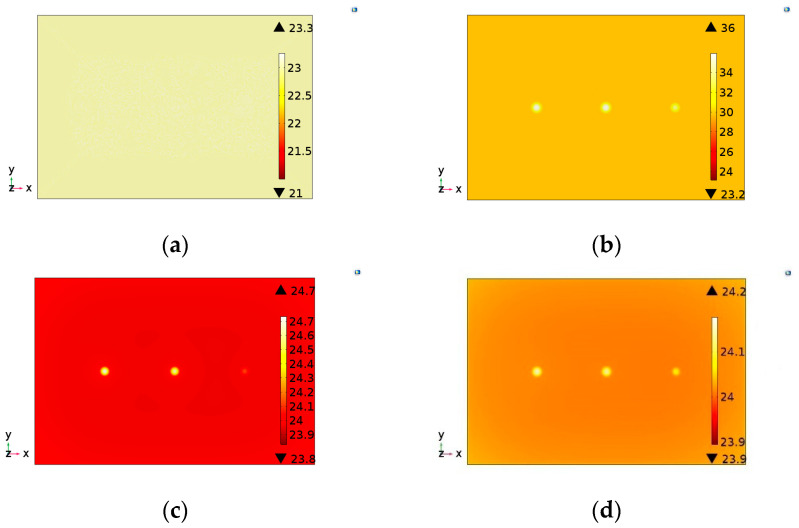
Images illustrating the temperature distribution on sample S2 obtained by the simulation model developed: (**a**) 0 s (at the moment that the lamps are turned on), (**b**) 10 s, (**c**) 20 s and (**d**) 30 s.

**Figure 8:**
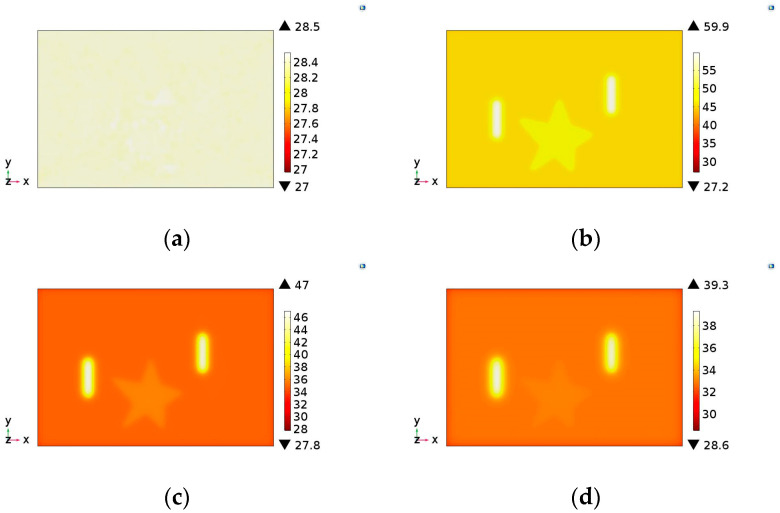
Images illustrating the temperature distribution on sample S3 obtained by the simulation model developed: (**a**) 0 s (at the moment that the lamps are turned on), (**b**) 10 s, (**c**) 20 s and (**d**) 30 s.

As in the experimental tests, the developed model presents as a result the evolution of the thermal distribution in the form of a sequence of images for each sample evaluated. In this model the image illustrating the thermal distribution interval was adopted of 1 s during the total testing time of 60 s. Thus, for each simulation performed, a sequence of 60 images was generated. Figure 6, Figure 7 and Figure 8 show the simulated images obtained for different observation times for the samples evaluated in this study.

**Figure 9:**
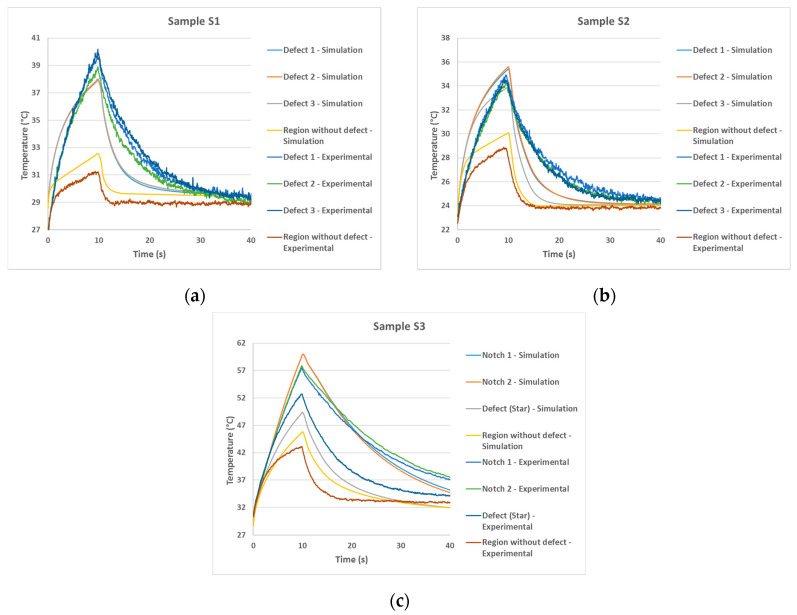
Comparison between temperature evolution obtained by simulation and experimentally for sample: (**a**) S1, (**b**) S2 and (**c**) S3.

Analyzing these images, all defects were detected, coherently with the experimental behavior. In addition, for sample S3, the adhesion failure (star) showed lower thermal contrast than the steel thickness loss (notches). This same behavior was observed in experimental results (Figure 5c). Therefore, the computational simulation model developed in this study showed a good correlation with thermograms obtained in the experimental tests. Even so, in addition to the comparison of obtained images, it was also considered necessary and important to evaluate the temperature evolution in defective and no defective regions obtained by the two methodologies (experimental and simulation). This comparison validates the efficiency of the developed model as a useful tool to virtually reproduce the PT inspection. Figure 9 shows the temperature evolution for the two methodologies in the evaluated samples.

Analyzing the graphs above, there is a small difference between the curves obtained by the simulation and the experimental ones. However, this difference is not considered significant since, in most of the results, this difference was smaller than the tolerable error of the thermographic camera reading (manufacturer considers error of ±2 °C ou ±2% of reading value). Moreover, this difference can be attributed to the influence of the external environment during the realization of the experimental tests and in the simulation there was no such influence since all external conditions were considered in the ideal condition, i.e., deterministic (noise free) environment.

So, it can be stated that the model created in this study was validated, for the inspection of samples representative of internal corrosion protective composite coatings for petrochemicals storage tanks, containing thickness loss defects due to localized corrosion (both holes defects and notches), and, to adhesion failure.

From this validation, the model becomes a useful tool for analyzing several factors in the thermal behavior of the samples in response to PT in a virtual environment, thus saving financial resources with the manufacture of samples, equipment and labor-hour. In this article, we chose to evaluate the influence of defects geometrical parameters on thermal contrast, such as depth and diameter. However, this model can also be used to analyze the influence of the power of the lamps used in the experiment, the thickness of the coating layer, the thickness of the substrate layer and the position of the defects in relation to the inspection surface, among other analyses. As each study of this influence involves the analysis of several parameters and for that, we would obtain a lot of data and the article would be very extensive, we chose to focus this article on the influence of geometrical parameters and inspection on the opposite side in thermal behavior (content described in the next item).

#### 4.2.1. Influence of Geometrical Parameters of Defects on Their Thermal Contrast Value

**Figure 10:**
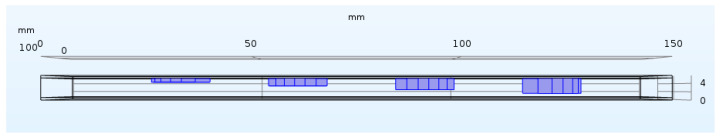
Cross section of the virtual solid illustrating defects of the same diameter with different depths (0.94, 1.88, 2.82 and 3.76 mm).

After the validation of the computational simulation model developed, it was considered important to evaluate the influence of some geometric parameters of defects, such as area and depth, on the thermal contrast value in order to estimate the limit of defection of the PT for inspection of these materials. For this purpose, a single virtual solid was constructed, with FBH defects with diameters of 5, 10 and 15 mm inserted. For each diameter, defects were made with depths of 0.94, 1.88, 2.82 and 3.76 mm (Figure 10). In this way, 12 different defects were made, with respect to the diameter and depth. The dimensions of this virtual solid corresponded to the same of the samples used in the experimental tests, considering a 0.6 mm thickness coating layer was applied on the both sides of the substrate. All defects were filled with iron oxide (Fe_3_O_4_) to represent the undercoating corrosion products.

**Figure 11:**
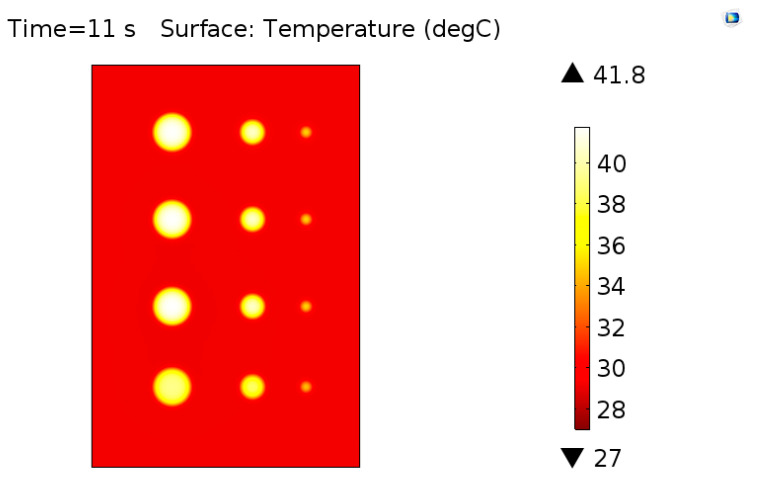
Image illustrating the thermal distribution on the sample obtained by the simulation at the time of the greatest thermal contrast.

**Figure 12:**
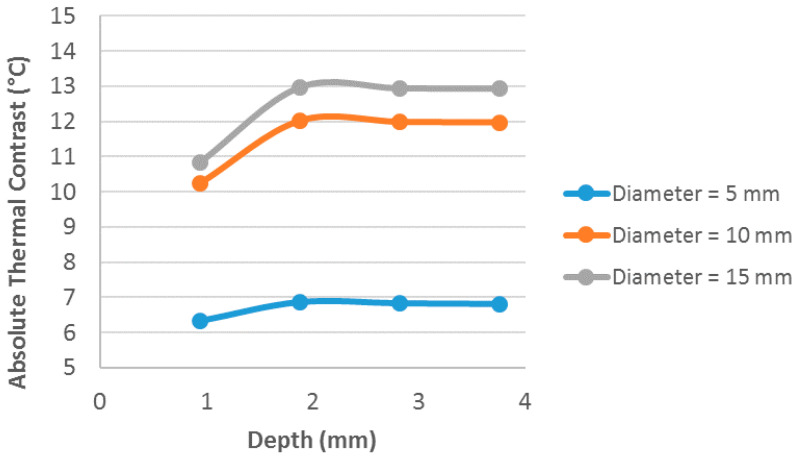
Analysis of the maximum thermal contrast behavior in relation to the depth of defects, with defects of 5, 10 and 15 mm diameter being evaluated.

Figure 11 shows the image of the surface of the sample at the time of the highest thermal contrast of defects. Analyzing this result, it is possible to observe that all defects were detected and there was an influence on the thermal contrast relative to both the defect area and depth. For a more detailed analysis, this influence was evaluated numerically through the maximum thermal contrast values obtained for each defect contained in the virtual solid and these data are also expressed in graphical form in Figure 12.

Through the analysis of these data, the depth of the defects generated a direct influence (increase), considering defects of the same diameter, in the thermal contrast value. The increase in the diameter of the defects generates an increase in the absolute thermal contrast.

#### 4.2.2. Inspection on the Opposite Surface of the Virtual Solid

Even with a thin layer of coating applied to both surfaces of the substrate, not allowing defects to be detected by a simple visual inspection, it was considered important to analyze the influence of defect parameters when they were inserted on the surface opposite to which is being inspected. In industry, it is not always possible to access the two surfaces of the equipment in which these materials are used, which makes such an analysis important to evaluate the detection capacity presented by technique is this condition.

For this analysis, a new virtual solid was built using the same characteristics of the defects in the previous items, however, in this case, due to the defects not being located on the front surface of the substrate, but under it, there is a thickness between the surface of the substrate and the surface where the defects starts, being called in this study the remaining thickness. This parameter can be calculated using the following equation:

(6) Remaining thickness =Substrate thickness−Defect depth

**Figure 13:**
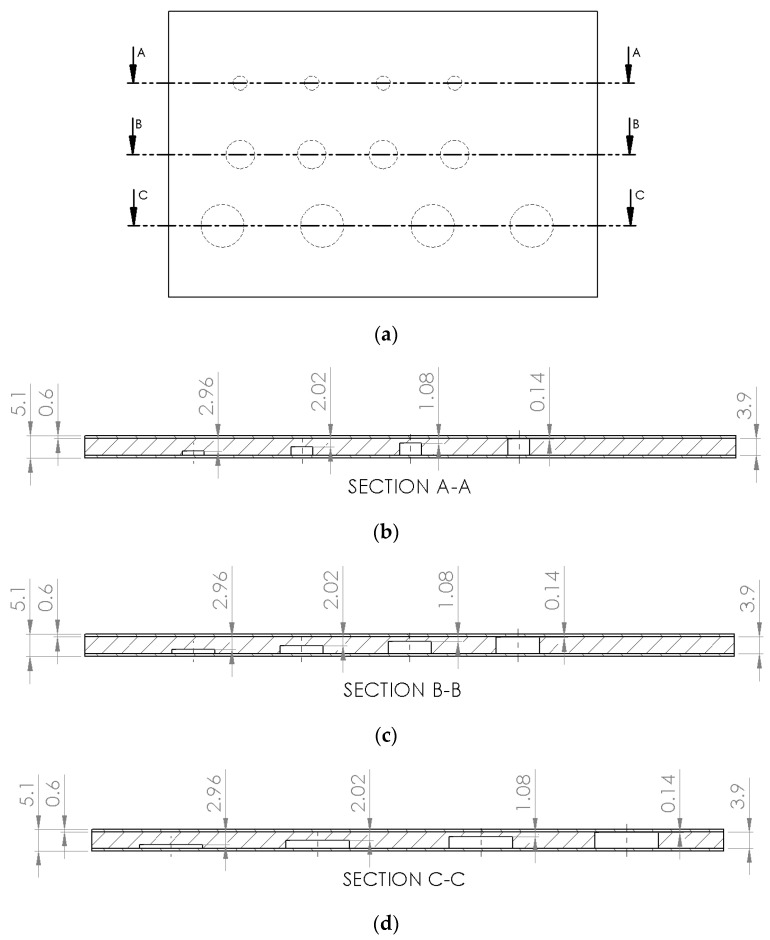
Inspection from the side opposite the defects: (**a**) top view of the virtual solid, (**b**) cut line for defects of 5 mm in diameter, (**c**) cut line for defects of 10 mm in diameter and (**d**) cut line for defects of 15 mm in diameter.

Table 4 shows the values of diameter and depth of the defects evaluated and their remaining thickness. Figure 13 shows the top view of the virtual solid used in this study and also the cut lines illustrating the positioning of the defects and the remaining thickness of them.

**Figure 14:**
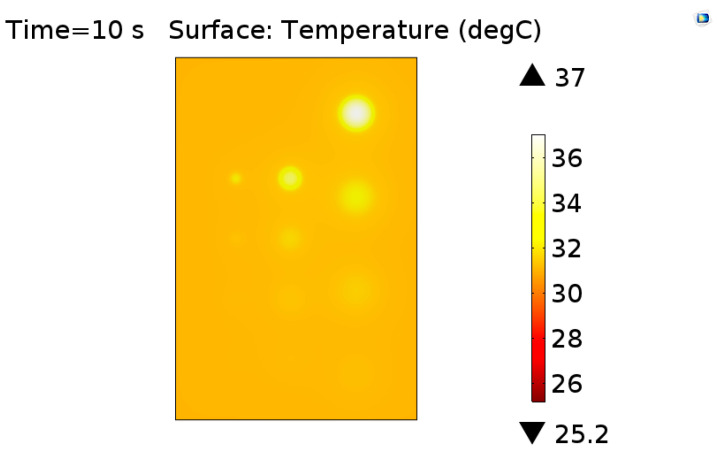
Image illustrating the thermal distribution of in the time of highest contrast, with inspection being performed on the opposite side of the defects.

From the result obtained by the computational simulation for the inspection performed on the surface opposite to the defects, as shown in Figure 14, it is possible to observe that only three defects had a level of thermal contrast sufficient to be detected. These defects corresponded to defects whose remaining thickness was equal to 0.14 mm and diameters of 5 mm, 10 mm and 15 mm. This result already provides the knowledge that defects with remaining thicknesses above 0.14 mm cannot be detected with this configuration adopted in the inspection due to the low thermal contrast presented. However, comparing the temperature intensity presented by each of these three defects shown in the image, it is possible to observe that as the diameter of the defect decreased, there was also a reduction in the temperature value presented by the defect, and consequently its thermal contrast also was reduced. This observed behavior, although inspection was being used on the side opposite to the defects, corroborated the behavior discussed in item 4.2.1 regarding the influence of the area (diameter) of the defect on the thermal contrast value when the inspection was carried out from the front side to the defects.

**Figure 15:**
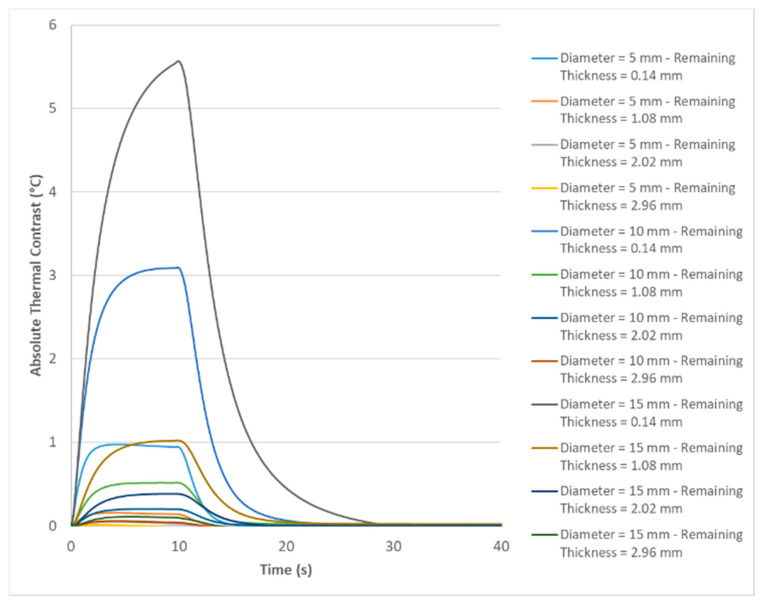
Evolution of absolute thermal contrast over time obtained by computational simulation for all defects contained in the sample (inspection performed on the opposite surface of the defects).

The values of maximum thermal contrast of the defects, shown in Table 5, were obtained from the curves shown in Figure 15. Analyzing these data of maximum thermal contrast, it is noted that for all three diameters evaluated, the increase in the remaining thickness of the defects generated a significant decrease of the maximum thermal contrast. This behavior can probably be explained by increasing the depth of penetration of the wave into the material until it finds the defect’s surface. Another aspect, already observed in the image obtained by the simulation, but which should be highlighted in the numerical analysis of this data set is that, considering defects of the same remaining thickness, the increase in area (diameter) gave a significant increase in the maximum thermal contrast value obtained, a reason that justifies the fact that the defect of 5 mm in diameter was not so easily identified in Figure 14 when compared to the defect of 15 mm in diameter, both with a remaining thickness of 0.14 mm.

**Figure 16:**
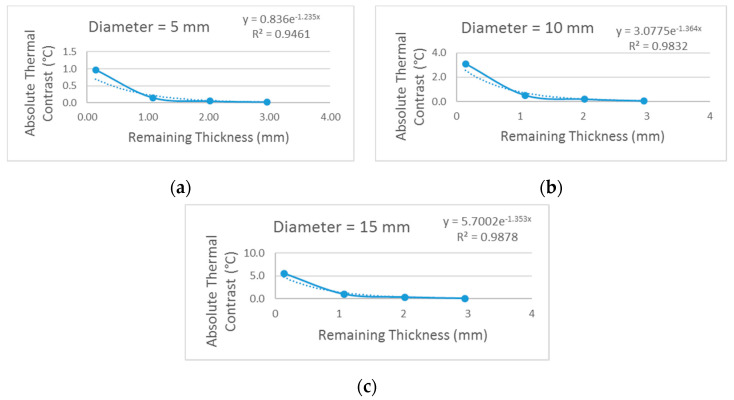
Absolute thermal contrast behavior for defects of: (**a**) 5 mm in diameter, (**b**) 10 mm in diameter and (**c**) 15 mm in diameter.

From the results obtained in Table 5, the reduction in the thermal contrast of the defects was evaluated as a function of the increase in the remaining thickness of the same, separating this analysis according to the diameter of the defects. These results are expressed in the graphs of Figure 16. In all three diameter sizes evaluated, a similar behavior was observed and in order to determine a function that best represents these data sets, adjustments were tested using functions linear, logarithmic, polynomial and exponential, among others. Adopting the value of R^2^ as a criterion for this evaluation, a parameter that represents the quality or efficiency of the model’s fit to the analyzed data, the best function obtained in all three defect diameters was the exponential function for presenting the largest R^2^ compared to other functions.

After defining the exponential function, the equation that best fit the data set for each of the defect diameters was obtained. From the knowledge of these equations it became possible to reconstruct the influence of the remaining thickness of defects on the absolute thermal contrast behavior. Thus, in this study we tried to estimate what is the limit of the remaining thickness of a defect so that it had a certain thermal contrast value that allowed its detection. For this, it was necessary to initially define a minimum thermal contrast value for detection of defects and in this study, 2 °C was considered for this parameter, since this value corresponded to the tolerable reading error by the thermal camera. Substituting this value in the equation to be the value of the parameter y, the value of x was calculated, corresponding to the remaining thickness value of the defect to be detected with such thermal contrast. Table 6 shows the remaining thickness limit value of the defects obtained for each evaluated diameter and the corresponding numerical parameters. Adopting the value of 2 °C as the minimum thermal contrast for the detection of defects, for defects of 5 mm in diameter it will not be possible to detect them. However, for defects whose diameter was 10 mm, inspection on the opposite side of the defects will be able to detect them up to a remaining thickness of 0.32 mm, that is, defects that were positioned up to 0.32 mm below the substrate surface will be detected, however defects with a diameter of 10 mm that were located in greater thicknesses will present thermal contrast values lower than 2 °C, a fact that makes it impossible to detect them according to the criterion chosen in this study. Considering the 15 mm diameter defects, this study estimated that it was possible to detect them if they are located up to a remaining thickness of 0.77 mm. Defects of 15 mm in diameter that were located in remaining thicknesses above 0.77 mm tended to have thermal contrast values below the chosen threshold of 2 °C, which prevented their detection.

The use of computational simulation to reproduce the thermographic inspection performed on the opposite side of the surface of the defects proved to be a very important study because through it, it became possible, initially, to confirm the detection capacity of the pulsed active thermography technique for some of the simulated defects in the virtual solid used in the study. Additionally, by reconstructing the behavior of the thermal contrast considering the remaining thickness of the defects for each diameter range evaluated in the study, it was estimated the limit of the remaining thickness of the defect for which it would be possible to obtain its detection using the pulsed modality.

## 5. Conclusions

In this study, the pulsed thermography (PT) was evaluated for detection of defects that may occur in steel with anticorrosive coatings. For this purpose, samples with controlled defects were manufactured, simulating undercoating localized corrosion and adhesion failures. All defects were machined with known dimensions and were not detectable by visual inspection.

According to the experimental results obtained, the PT was able to detect all defects (localized undercoating corrosion and adhesion loss). No significant difference in thermal contrast was observed in relation to the type of holes used in the defects (FBH and conical holes). Considering the defect detection, there was no influence on it whether the localized corrosion was filled or not with iron oxide. This is an original result hardly approached in the literature.

The efficiency of the computational simulation model developed in this study was evaluated and there was a good correlation both in the images of the surface temperature distribution and in thermal behavior over time between the defective and non-defective regions both for experimental and simulated data. Moreover, the simulation reproduced the thermal contrast between the two types of defects, as presented in the experimental results. From this model it was possible to numerically evaluate the influence of geometrical parameters (diameter and depth) of the defects in their absolute thermal contrast. Additionally, from the simulation performed on the opposite surface of the defects, through some adjustments in the model configuration, it was possible to observe that the pulsed thermography technique presented a defect detection limit of 10 mm in diameter with remaining thicknesses up to 0.32 mm and for defects of 15 mm in diameter, the remaining thickness limit observed was 0.77 mm.

## Figures and Tables

**Table 1:** Defects in samples S1, S2 and S3.

Defects	S1 *		S2 *		Defects	S3		
	Diam. (mm)	Depth (mm)	Diam. (mm)	Depth (mm)		Width (mm)	Length (mm)	Depth (mm)
hole 1	5.65	3.43	4.89	3.51	notch 1	5.99	25.02	3.26
hole 2	5.56	2.46	4.98	2.46	notch 2	5.96	24.99	3.15
hole 3	5.53	1.47	4.64	1.37	notch 3 (star)	51.25	52.22	0.05

* holes filled with Fe_3_O_4_.

**Table 2:** Specifications of the coatings.

Sample	Coating	Dry Coating Thickness (µm)
S1	epoxy phenolic reinforced with glass flake	582 ± 125
S2		670 ± 140
S3	epoxy reinforced with ceramic flakes	719 ± 33

**Table 3:** Materials chosen to represent each sample component.

Component	S1	S2	S3
Substrate	Steel AISI 4340		
Coating	Filled Epoxy Resin		
Defects	Fe_3_O_4_	Fe_3_O_4_	Air

**Table 4:** Remaining thickness values of the defects inserted in the virtual solid for inspection on the opposite surface.

Defects		
Diameter (mm)	Depth (mm)	Remaining Thickness (mm)
5	3.76	0.14
	2.82	1.08
	1.88	2.02
	0.94	2.96
10	3.76	0.14
	2.82	1.08
	1.88	2.02
	0.94	2.96
15	3.76	0.14
	2.82	1.08
	1.88	2.02
	0.94	2.96

**Table 5:** Maximum values of absolute thermal contrast of the defects present in the virtual solid, with inspection being carried out on the opposite side to the defects.

Defects		Maximum Absolute Thermal Contrast (°C)
Diameter (mm)	Remaining Thickness (mm)	
5	0.14	0.97
	1.08	0.16
	2.02	0.05
	2.96	0.03
10	0.14	3.09
	1.08	0.52
	2.02	0.20
	2.96	0.06
15	0.14	5.56
	1.08	1.02
	2.02	0.38
	2.96	0.11

**Table 6:** Limit values for the remaining thickness of the defects for their detection, with inspection being carried out on the side opposite to the defects.

Diameter of Defects (mm)	R^2^	Equation	Minimum Thermal Contrast (y)	Remaining Thickness Required for Detection (x)
5	0.9461	y = 0.836 × 10 ^−1.235x^	2 °C	Not detectable
10	0.9832	y = 3.0775 × 10 ^−1.364x^	2 °C	0.32 mm
15	0.9878	y = 5.7002 × 10 ^−1.353x^	2 °C	0.77 mm
